# Cost-Effectiveness of Nutrient Supplementation in Cancer Survivors

**DOI:** 10.3390/cancers13246276

**Published:** 2021-12-14

**Authors:** Amy L. Shaver, Theresa A. Tufuor, Jing Nie, Shauna Ekimura, Keri Marshall, Susan Hazels Mitmesser, Katia Noyes

**Affiliations:** 1 Department of Epidemiology and Environmental Health, Division of Health Services Policy and Practice, School of Public Health and Health Professions, University at Buffalo, Buffalo, NY 14214, USA; amyshave@buffalo.edu (A.L.S.); tatufuor@buffalo.edu (T.A.T.); jingnie@buffalo.edu (J.N.); 2 Department of Pharmacy Practice, School of Pharmacy and Pharmaceutical Sciences, University at Buffalo, Buffalo, NY 14214, USA; 3 Pharmavite LLC, West Hills, CA 91304, USA; sekimura@pharmavite.net (S.E.); kmarshall@pharmavite.net (K.M.); smitmesser@pharmavite.net (S.H.M.)

**Keywords:** vitamin, mineral, nutrition, cancer, nutritional deficiency, supplementation, cost-effectiveness, QOL

## Abstract

**Simple Summary:**

Cancer patients and cancer survivors are at risk for malnutrition from both their disease and its treatment. Many cancer survivors use dietary supplementation without informing their doctors. The goal of this study was to examine the prevalence and cost-effectiveness of dietary supplementation in a nationally representative sample of cancer survivors in the U.S. by looking at intake and hospitalization records. Adequate nutrition is a cost-effective way to promote well-being More research needs to be carried out so providers can offer the best nutrition to the right patients at the right time.

**Abstract:**

Cancer patients are at risk for malnutrition; the aim of this study was to provide a cost-effectiveness analysis of dietary supplementation in cancer survivors. We estimated prevalence of supplementation, hospitalization rates, quality of life (QOL), cost of care and mortality among cancer survivors. We built a decision analytic model to simulate life-long costs of health care and supplementation and QOL among cancer survivors with and without supplementation. Cost of supplements was derived from national pharmacy databases including single- and multivitamin formularies. One-way and probabilistic sensitivity analysis were performed to evaluate the robustness of the incremental cost-effectiveness ratio (ICER) to changes in supplementation costs and duration. The study cohort represented the national cancer survivor population (average age 61 years, 85% white, 52% male, and 94% insured). Hospitalization rates for supplement users and non-users were 12% and 21%, respectively. The cost of hospitalization was $4030. Supplementation was associated with an additional 0.48 QALYs (10.26 vs. 9.78) at the incremental cost of $2094 ($236,933 vs. $234,839) over the remaining lifetime of survivors (on average 13 years). Adequate nutrition provides a cost-effective strategy to achieving potentially optimum health. Further studies are needed to determine the effects of specific nutrient doses and supplementation on long-term outcomes per cancer type.

## 1. Introduction

Adequate nutrition throughout the continuum of care is important for optimizing short- and long-term clinical and economic outcomes of all patients [1]. Evidence demonstrates that nutrition interventions in the general healthy population can serve as effective preventive as well as therapeutic measures [2]. Despite $12.8 billion a year spent by Americans on dietary supplements (DS), limited data exist on the cost-effectiveness and long-term economic sustainability of these nutrition interventions and approaches [3].

Cancer patients are especially at risk for malnutrition as cancer has a serious negative impact on patient ability to consume and absorb nutrients and, hence, their functioning [4]. A systematic review of studies from 1999 to 2006 found that DS use is highly prevalent among the general population and cancer survivors [5,6]. In the general US population, 50% reported using DS and 33% used multivitamins. In contrast, 64–81% of cancer survivors reported using DS and 26–77% reported using a multivitamin, with significant variation by cancer type, sex and education [5]. Several studies indicated that between one-third and two-thirds of cancer patients do not discuss their DS use with their healthcare providers [7,8]. 

Hospitalization is a key marker of cancer quality of care and stands out as the main driver of cancer-related health care spending (aside from primary tumor resection) among cancer patients [9]. In Western countries, cancer treatment is performed mainly in the outpatient and ambulatory settings. Due to predominately ambulatory treatment regimens, an inpatient admission during cancer treatment or survivorship phases is usually an indicator for a complication or adverse event and possibly the result of poor quality of care. Accordingly, Centers for Medicare and Medicaid used hospital admissions in cancer patients as a ground for decreasing provider reimbursement in the Oncology Care Model [10].

DS use among cancer survivors is controversial. For cancer patients currently receiving treatment, the recommendation is to use caution with DS due to possible interactions with chemotherapy. The gold standard is to achieve adequate nutrition intake through food sources, although nutrient adequacy is not often met for a variety of reasons, including the side effects of treatment [8,11,12].

Adult cancer survivors spend $6.8 billion on DS annually [6]. Given the significant variation in prices and formulation of DS, lack of evidence on cost-effectiveness of DS use for cancer survivors in the United States may limit patients’ ability to make educated decisions about DS risks and benefits. Using national population health survey and marketplace costs data with a broad policy perspective, this study seeks to close this gap for both the general public and for their providers. We performed a population-level analysis according to ISPOR’s good modeling practices exploring the impact of long-term DS use on quality adjusted life years and DS potential for cost-effectiveness in a cancer population [13,14].

## 2. Materials and Methods

Data on nutrient intake and other patient-level information were obtained from the 2011–2012 cycle of the National Health and Nutrition Examination Survey (NHANES). The NHANES is a nationally representative, cross-sectional survey conducted by the National Center for Health Statistics (NCHS) used to monitor the health and nutritional status of non-institutionalized individuals in the U.S. Nationally representative samples are drawn each year from the US population using a stratified multistage probability sampling method. 

Hospitalization cost was estimated from the Medical Expenditure Panel Survey (MEPS) [15]. The MEPS provides estimates of healthcare use, expenditures, source of payment, and health insurance coverage. Total inpatient costs are a sum of payments for care provided during inpatient hospitalizations and include out-of-pocket payments and payments by private insurance, Medicaid, Medicare, and other sources. Cancer survivors were identified as those who answered ‘yes’ to the question, “Have you ever been told you had cancer or a malignancy?” This included both those who were actively under a physician’s care and those who had long since overcome their malignancy.

All adults aged 20 years and older who answered yes to having ever been told they had cancer from the 2011–2012 cycle were included in the analysis. Adults with missing values of dietary and supplement intake were excluded as were those who did not answer both days of dietary intake questionnaires. The final weighted sample of participants was 14,364,981 (Table 1). 

NHANES participants were asked to respond to the medical condition questionnaire at their homes by a trained interviewer. Questioned varied based on the respondent sex (for conditions related to specific anatomy) and age. Participants were asked if they had ever been told they had cancer, arthritis, diabetes, congestive heart failure (CHF), chronic obstructive pulmonary disease (COPD), or hypertension.

Participant demographics (age, sex, and race) were self-reported during NHANES interviews. Social characteristics were also provided by the participants and included smoking status (current, former, and never smoker); level of education (≤high school graduate, some college, and ≥bachelor’s degree). Participants answered whether they were US citizens. Socioeconomic status was assessed in terms of income to poverty ratio (IPR) (<100%, 100 to <200%, 200 to <300%, and ≥300%) and health insurance (private, Medicare, other or none). Body mass index (BMI) was measured as part of NHANES anthropometric examination and was utilized to calculate obesity (BMI ≥ 30).

All NHANES participants are eligible to participate in two 24-hour dietary recall interviews. The first interview is performed in the Mobile Examination Center with a second telephonic interview following 3–10 days later [16,17]. The recall interviews are conducted by trained NCHS interviewers. If a participant indicated vitamin or DS use, the interviewer then examines the dietary supplement container and enters the product name and strength into the Computer-Assisted Personal Interviewing (CAPI) system. Trained NCHS nutritionists discern the exact product reported by participants and calculate daily average intake of nutrients based on the previous 30 days’ food, dietary supplement, and antacid intake. Nutrient intake is subsequently broken down by source (food or DS). 

Mortality outcomes were obtained for each participant through linkage to the National Death Index through 31 December 2015 [18]. The life expectancy for those who did not die was estimated based on age- and sex-specific life expectancy tables for cancer patients derived based on SEER population-based data in the US and similar datasets in the UK and Italy [19,20,21,22]. The average weighted life expectancy for the study cohort was 13 years (from 61 years at the time of the interview to 74 years of age).

Quality adjusted life years (QALY) is the main outcome when assessing cost-effectiveness of health interventions [23,24,25]. To estimate QALY, one needs to assess simultaneously two outcomes: average population life expectancy and quality of life (QOL) or health utility in each study arm. Participants in the NHANES were asked to rate their health on a scale from 1 (excellent health) to 5 (poor health). Participants were also asked to indicate on how many days in the past 30 days their physical health was not good; they were asked to indicate the same for their mental health. 

**Figure 1:**
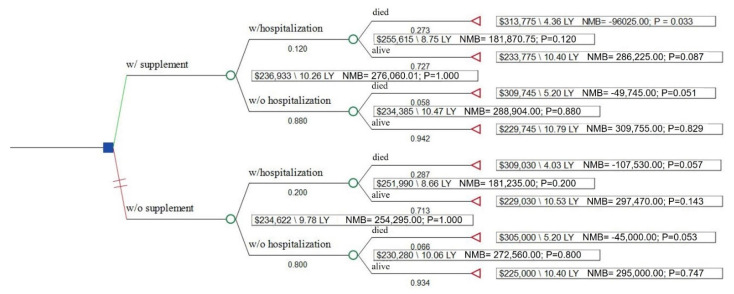
Decision tree analysis of cost-effectiveness of dietary supplementation. LY, life years; NMB, net monetary benefit.

The EQ-5D is a health utility instrument necessary to the calculation of quality adjusted life years (QALY) used in the calculation of an incremental cost-effectiveness ratio (ICER) [23,24,25]. The nominal range of EQ-5D scores is from 0 to 1, with 0 representing death and 1 representing perfect health (scores below 0 for worse than death have been reported). To estimate health utility for each care pathway presented in the decision analytic model (Figure 1), the Healthy Days measure was converted to a EQ-5D value using an algorithm developed and validated by Jia et al. [26].

To minimize heterogeneity among treatment protocols for different cancer types, outpatient health care costs were divided into three timeframes: first year following cancer diagnosis, continuing phase, and last year of life. Estimates for each of these timeframes were based on the estimates generated using MEPS and SEER Medicare data [27,28]. Hospitalization costs for cancer survivors were estimated using 2011–2012 MEPS data. The cost-effectiveness analysis was performed from the societal perspective (including direct and indirect costs).

Costs of DS were estimated using online retail prices for the leading brands that produce DS (Nature Made, CVS, Walmart, and Walgreens private-labeled brands) as of June 2020 [29]. To estimate unit cost of multivitamin products, we selected the products that included at least 11 out of the 16 core nutrients of interest. Costs of dietary supplements not commonly included in the multivitamin products were estimated separately using single-nutrient products. Daily costs of DS were calculated based on the recommended daily intake amount [30,31,32].

To minimize patient self-selection bias as a result of unobserved selection to use dietary supplements, NHANES patients with and without DS use were matched using 1:1 propensity score approach (on age, sex, race, income, level of education, comorbidities, citizenship status, insurance coverage, smoking status, survey cycle, and if they had a regular health care provider). The matching resulted in two non-different samples. Analyses were conducted using appropriate survey weights in SAS 9.4.

The decision analytic model was built to simulate life-long costs and QOL among cancer survivors with and without supplementation using TreeAge Healthcare software (2020 R1.2). Costs included cost of DS for the rest of patient life expectancy, cost of acute care inpatient hospitalization, and cost of care throughout the cancer continuum which included both direct health care costs as well as the indirect cost of lack of productivity. One-way and probabilistic sensitivity analyses were performed to evaluate the robustness of the incremental cost-effectiveness ratio (ICER) to changes in supplementation costs and duration (Table 2) using parameter distributions recommended by Sanders et al. [24].

## 3. Results

### 3.1. Population Characteristics

The study sample was representative of the national population of individuals with cancer diagnosis (Table 1). Participants were on average 61 years of age, 85% white and predominantly male. A majority (70.3%) had at least some college education; over 40% were obese. Most had a regular health care provider (93.7%) and the vast majority had some form of insurance (94.4%). More than two-thirds of the sample (68%) reported using some form of vitamin or DS within the previous 30 days. 

### 3.2. Health and Economic Outcomes

The probability of hospitalization among those reporting DS use was 12.0% vs. 20.7% among those not reporting DS use. The probability of dying during the follow-up period was 5.8% among DS users without a hospitalization in the past year and 27.3% in DS users with a hospitalization. Among non-DS users, the probability was 6.6% in non-hospitalized cancer patients and 28.7% among those with a hospitalization. Average health utility, by hospitalization and DS status, is reported in Table 2. Cancer patients who have had a hospitalization during the prior year had over 6-fold higher odds of all-cause mortality compared to those who have not had a hospitalization (OR 6.47; 95% CI 0.82, 50.82; *p* = 0.07 for patients without DS use and OR 6.04; 95% CI 1.00, 36.38; *p* = 0.05 for those who used DS) (Table S1).

Average annual cost of all-cause hospitalization among cancer survivors was $4030 (95% CI $3310; $4749). Daily cost of DS use was $1.00 ($0.01–$10.00). Health care costs over the initial year following cancer diagnosis were $60,000 (ranges $20,000–$100,000); continuing years $15,000 (range $1000–$30,000); and for the last year of life $80,000 (range $60,000–$200,000) (Table S2).

### 3.3. Cost-Effectiveness and Sensitivity Analyses

In the base case, DS use in cancer survivors was cost-effective ($4362/QALY)m generating an additional 0.48 QALYs (10.26 vs. 9.78) at the incremental cost of $2094 ($236,933 vs. $234,839) over the remaining lifetime of cancer survivors (on average 13 years) (Table 3). 

**Figure 2:**
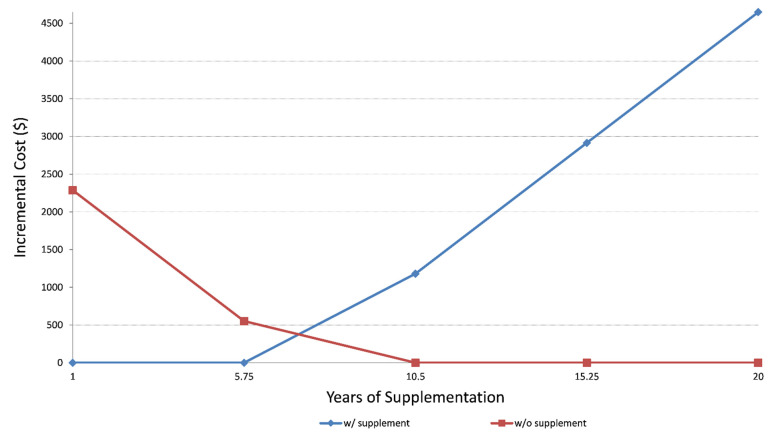
Sensitivity analysis showing incremental cost and years of supplementation. The probabilistic sensitivity analysis (Figure 3) demonstrates that the large portion of the CE ellipse in quadrant II is located below the cost-effectiveness threshold. This indicates that variation of the modeling parameters is unlikely to reverse the study conclusion about cost-effectiveness of DS use in cancer survivors.

Sensitivity analyses utilizing lower and upper bounds of life expectancy (1 year to 20 years) and incremental costs (from $0 to $4500) (Figure 2) demonstrated that after six years of supplement use, the benefit of lower hospitalization rates among DS users outweighs the cost of dietary supplementation.

## 4. Discussion

This study is based on the current real-life patterns of DS use by cancer survivors using nationally representative data. Cancer survivors who used DS had a significantly lower rate of hospitalizations and reported higher quality of life (QOL) compared to survivors who did not use any dietary supplements. Life-long supplementation use was cost-effective at $4362/QALY. These findings were robust to changes in most model parameters.

Previous studies have shown that receiving a cancer diagnosis leads to lifestyle changes, including changes in nutrition, through diet and DS [33,34]. Reasons for DS use vary but the most commonly reported are to improve immune function, prevent deficiency, and prevent disease recurrence [35]. Cancer patients require special attention both during and following treatment since both cancer [36,37] and its treatment can impair nutrient absorption [38,39].

The evidence on benefits of DS use both during and after cancer treatment is conflicting. Several studies report increased mortality with increased use of B12 and iron during treatment but no increase in mortality with multivitamins [40]. In a large observational study, DS was linked with increased mortality when used by those who concurrently had a nutritionally poor diet [41]. In the Molisani study, serum D deficiency was independently associated with risk of hospitalization for heart failure [42]. This is in accord with our study which showed a decreased risk of all-cause hospitalization in DS users. A Women’s Health Initiative study showed little to no effect on DS on the risk of cancer recurrence, cardiovascular disease, or mortality [43]. In an extensive review of DS in cancer survivors, benefits were dependent on factors including cancer type and treatment, consistency in use and overall health status [44]. Paur et al. found a decrease in prostate-specific antigen with high plasma levels of lycopene [45]. Selenium was associated with decreased risk of prostate cancer in men as well as decreased breast cancer mortality in women [46,47]. Dietary vitamin C was associated with decreases in both total and breast cancer-specific mortality [48]. The American Cancer Society recommends that DS should not exceed 100% of recommended daily values in cancer patients, while outside of deficiency, the European Society for Clinical Nutrition and Metabolism recommends against DS [49,50]. However, the nutrition status of cancer patients is oftentimes insufficient due to both their disease state and treatment [51,52,53].

Our study has some limitations driven mainly by the limitations of the available data sources. Our study population was predominately white, males in their early 60s and of a higher socioeconomic status, which is consistent with the national cancer statistics but may not be generalizable to low income populations [54,55]. Neither costs nor outcomes were adjusted for cancer stage or number of years since diagnosis nor if participants are still in active treatment. Mortality is relatively rare in the NHANES sample and it would require a significantly larger sample to precisely estimate it. The hybrid modelling approach used for this study allowed the combination of data from multiple sources. The main benefit of hybrid economic modelling is to explore the relationship between different parameters (e.g., duration of use and incremental costs of care). It is not used as a substitute to randomized controlled trial that provides the best estimate of DS effectiveness [23,24,25]. Due to small sample size, analysis by cancer type was not possible within the NHANES or the MEPS. Our analysis only included dietary supplementation and no other forms of supplementation such as total parenteral nutrition which was not included in either survey. Finally, no data were available to estimate hospitalization cost by DS use status.

## 5. Conclusions

There are several important implications for current practice related to this study’s findings. The importance of nutritional assessment for both active cancer patients and long-term survivors cannot be underestimated. In particular, for cancer types where the evidence is conclusive, nutritional therapy for cancer survivors should involve education on daily food intake, dietary supplementation self-management and prescription nutrition therapy where warranted. More research is needed on the role and duration of DS in cancer patients to determine the level of dose response. There is a need to clarify the role DS may play on various comorbidities and late side effects in the cancer survivor such as cardiovascular disease, diabetes, and other metabolic disorders. Further, there is a need to elucidate the mechanism of DS effect on immune system modulation affecting cancer survivors. Given the overall cost-effectiveness of DS, there is a need for better provider education about how to talk with cancer survivors about their nutrient status and filling nutrient gaps through both food and supplements. Immune-supportive supplementation may prove to be a clinically effective and important tool that is accessible via telemedicine.

## Figures and Tables

**Figure 3:**
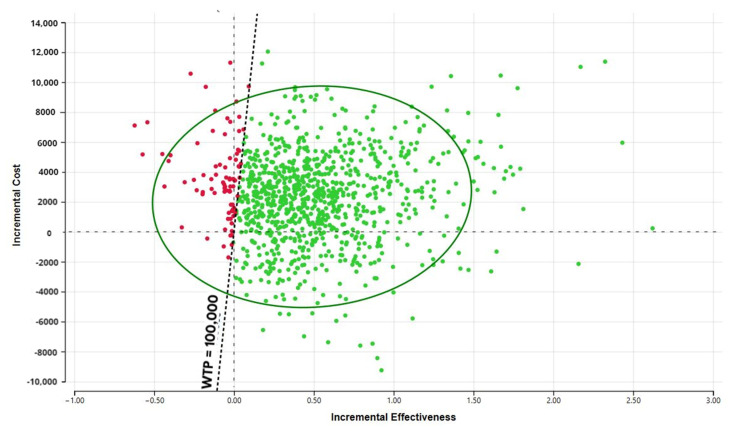
Probabilistic sensitivity analysis.

**Table 1:** Population characteristics by prior 30 day dietary supplement use.

Characteristic	No Supplement Use *n* = 7,097,880	Supplement Use *n* = 7,267,101	*p*
Age	60.5 {1.3}	61.6 {1.3}	0.52
Sex			
Male	3,791,028 (53.4)	3,759,878 (51.7)	0.81
Female	3,306,851 (46.6)	3,507,222 (48.3)	
Race			
Mexican American	137,969 (1.9)	159,221 (2.2)	0.97
Other Hispanic	176,846 (2.5)	150,702 (2.1)	
NH White	6,090,800 (85.8)	6,196,220 (85.3)	
NH Black	443,511 (6.2)	429,859 (5.9)	
Other	248,754 (3.5)	331,098 (4.6)	
Education			
≤High School Graduate	2,286,161 (32.2)	1,982,235 (27.3)	0.61
Some College	2,800,442 (39.5)	2,786,318 (38.3)	
≥ Bachelor’s Degree	2,011,276 (28.3)	2,498,547 (34.4)	
Income to Poverty Ratio			
<100% FPL	850,849 (12.0)	716,112 (9.9)	0.63
100 to <200% FPL	1,516,662 (21.4)	1,440,526 (19.8)	
200 to <300% FPL	702,935 (9.9)	683,737 (9.4)	
>=300% FPL	3,051,271 (43.0)	3,807,709 (52.4)	
Missing	976,163 (13.8)	619,017 (8.5)	
Comorbidities			
Arthritis	3,966,058 (55.9)	3,444,156 (47.4)	0.26
Diabetes	1,281,946 (18.1)	1,586,416 (21.8)	0.49
CHF	349,043 (4.9)	417,474 (5.7)	0.75
COPD	1,235,382 (17.4)	768,467 (10.6)	0.17
HTN	3,777,368 (53.2)	3,737,143 (51.4)	0.79
Obese	3,016,614 (42.5)	2,923,929 (40.2)	0.77
US Citizen	6,602,817 (93.0)	6,857,931 (94.4)	0.49
Regular Health Care Provider	6,687,467 (94.2)	6,774,950 (93.2)	0.80
Smoker			
Current	1,846,695 (26.0)	1,777,151 (24.5)	0.52
Former	2,182,592 (30.7)	2,766,162 (38.1)	
Never	3,068,593 (43.2)	2,723,787 (37.5)	
Insurance			
Private	2,746,533 (38.7)	2,892,542 (39.8)	0.50
Medicare	2,603,265 (36.7)	2,634,374 (36.3)	
Other	1,184,830 (16.7)	1,499,233 (20.6)	
None	563,252 (7.9)	240,952 (3.3)	
Quality of life			
Physical health poor	6.7 [1.2]	3.6 [1.1]	0.09
Mental health poor	4.9 [1.1]	3.6 [1.1]	0.36
EQ5D	0.8 [0.0]	0.9 [0.0]	0.01

Data are presented as the mean {SD} or [SE] and frequency (%). CHF, congestive heart failure; COPD, chronic obstructive pulmonary disease HTN, hypertension; FPL, federal poverty limit; NH, non-Hispanic.

**Table 2:** Distributions and parameters for the probabilistic sensitivity analysis.

Variable Name			Distribution Type	Parameters Mean (St. Dev)
Probability Hospitalization				
With supplement use			Beta	0.12 (0.05)
No supplement use			Beta	0.20 (0.05)
Probability mortality				
Supplement Use	Hospitalized			
X	X		Beta	0.273 (0.05)
X			Beta	0.058 (0.01)
	X		Beta	0.287 (0.05)
			Beta	0.066 (0.01)
Years survival			Gamma	13 (5)
Cost of hospitalization			Gamma	4030 (2000)
Cost of health care				
Initial year			Gamma	60,000 (20,000)
Continuing years			Gamma	15,000 (5000)
Last year of life			Gamma	80,000 (30,000)
EQ5D				
Supplement use	Hospitalized	Died		
X	X		Beta	0.80 (0.02)
X	X	X	Beta	0.67 (0.02)
X			Beta	0.83 (0.02)
X		X	Beta	0.80 (0.02)
	X		Beta	0.81 (0.02)
	X	X	Beta	0.62 (0.02)
			Beta	0.80 (0.02)
		X	Beta	0.80 (0.02)

**Table 3:** Incremental cost-effectiveness ratios and health outcome, by supplement use.

Measure (Average Per Patient)	No Supplement Use	Supplement Use
Health outcome		
QALYs	9.78	10.26
ICER		
Base case costs	$234,839	$236,933
ΔCost/ΔQALY		4362
Simulation: 1 year life expectancy	$54,839	$52,553
ΔCost/ΔQALY		dominant
Simulation: 20 year life expectancy	$339,839	$344,488
ΔCost/ΔQALY		6348

## Data Availability

The data that support the findings of this study were derived from the following resources available in the public domain: 2011–2012 NHANES cycle dietary data, demographics data, and questionnaire data [56,57]. The data that support the findings of this study were also derived from the following resources available in the public domain: the 2011 and 2012 Full Year Consolidated Data Files [58,59].

## Supplementary Materials

The following are available online at https://www.mdpi.com/article/10.3390/cancers13246276/s1, Table S1: Odds of mortality according to supplementation use and previous hospitalizations; Table S2: Estimated average (per person) ten-year costs, by type and supplement use; Table S3: Amount of nutrients per daily dose/serving size and cost range; Tables S4 and S5: Amount of multivitamin and single-nutrient products per daily dose/serving size and cost range.

## References

[1] Alderman H., Behrman J.R., Hoddinott J. (2007). Economic and nutritional analyses offer substantial synergies for understanding human nutrition. J. Nutr..

[2] Bowen D.J., Beresford S.A. (2002). Dietary interventions to prevent disease. Annu. Rev. Public Health.

[3] Nahin R.L., Barnes P.M., Stussman B.J. (2016). Expenditures on Complementary Health Approaches: United States, 2012.

[4] PDQ Supportive Palliative Care Editorial Board (2002). Nutrition in Cancer Care (PDQ^®^): Patient Version. PDQ Cancer Information Summaries [Internet].

[5] Velicer C.M., Ulrich C.M. (2008). Vitamin and mineral supplement use among US adults after cancer diagnosis: A systematic review. J. Clin. Oncol..

[6] John G.M., Hershman D.L., Falci L., Shi Z., Tsai W.-Y., Greenlee H. (2016). Complementary and alternative medicine use among US cancer survivors. J. Cancer Surviv..

[7] Du M., Luo H., Blumberg J.B., Rogers G., Chen F., Ruan M., Shan Z., Biever E., Zhang F.F. (2020). Dietary Supplement Use among Adult Cancer Survivors in the United States. J. Nutr..

[8] Pouchieu C., Fassier P., Druesne-Pecollo N., Zelek L., Bachmann P., Touillaud M., Bairati I., Hercberg S., Galan P., Cohen P. (2015). Dietary supplement use among cancer survivors of the NutriNet-Santé cohort study. Br. J. Nutr..

[9] Whitney R.L., Bell J.F., Tancredi D.J., Romano P.S., Bold R.J., Wun T., Joseph J.G. (2019). Unplanned Hospitalization Among Individuals With Cancer in the Year After Diagnosis. J. Oncol. Pract..

[10] CMS Oncology Care Model. https://innovation.cms.gov/innovation-models/map#model=oncology-care-model.

[11] Rock C.L., Doyle C., Demark-Wahnefried W., Meyerhardt J., Courneya K.S., Schwartz A.L., Bandera E.V., Hamilton K.K., Grant B., McCullough M. (2012). Nutrition and physical activity guidelines for cancer survivors. CA Cancer J. Clin..

[12] American Cancer Society Risks and Side Effects of Dietary Supplements. https://www.cancer.org/treatment/treatments-and-side-effects/complementary-and-alternative-medicine/dietary-supplements/risks-and-side-effects.html.

[13] Weinstein M.C., O’Brien B., Hornberger J., Jackson J., Johannesson M., McCabe C., Luce B.R. (2003). Principles of good practice for decision analytic modeling in health-care evaluation: Report of the ISPOR Task Force on Good Research Practices—Modeling Studies. Value Health.

[14] Husereau D., Drummond M., Petrou S., Carswell C., Moher D., Greenberg D., Augustovski F., Briggs A.H., Mauskopf J., Loder E. (2013). Consolidated health economic evaluation reporting standards (CHEERS) statement. Int. J. Technol. Assess. Health Care.

[15] Cohen J.W., Cohen S.B., Banthin J.S. (2009). The medical expenditure panel survey: A national information resource to support healthcare cost research and inform policy and practice. Med. Care.

[16] CDC 2011-2012 Data Documentation, Codebook, and Frequencies: Dietary Supplement Use 30-Day—Total Dietary Supplements (DSQTOT_G). https://wwwn.cdc.gov/Nchs/Nhanes/2011-2012/DSQTOT_G.htm.

[17] CDC 2015-2016 Data Documentation, Codebook, and Frequencies: Dietary Supplement Use 30-Day—Total Dietary Supplements (DSQTOT_I). https://wwwn.cdc.gov/Nchs/Nhanes/2015-2016/DSQTOT_I.htm.

[18] NCHS (2018). The Linkage of National Center for Health. Statistics Survey Data to the National Death Index–2015 Linked Mortality File (LMF): Methodology Overview and Analytic Considerations.

[19] Arias E. (2015). United States Life Tables, 2011. Natl. Vital Stat. Rep..

[20] Syriopoulou E., Bower H., Andersson T.M., Lambert P.C., Rutherford M.J. (2017). Estimating the impact of a cancer diagnosis on life expectancy by socio-economic group for a range of cancer types in England. Br. J. Cancer.

[21] Capocaccia R., Gatta G., Dal Maso L. (2015). Life expectancy of colon, breast, and testicular cancer patients: An analysis of US-SEER population-based data. Ann. Oncol..

[22] Botta L., Dal Maso L., Guzzinati S., Panato C., Gatta G., Trama A., Rugge M., Tagliabue G., Casella C., Caruso B. (2019). Changes in life expectancy for cancer patients over time since diagnosis. J. Adv. Res..

[23] NICE (2013). Guide to the Methods of Technology Appraisal.

[24] Sanders G.D., Neumann P.J., Basu A., Brock D.W., Feeny D., Krahn M., Kuntz K.M., Meltzer D.O., Owens D.K., Prosser L.A. (2016). Recommendations for Conduct, Methodological Practices, and Reporting of Cost-effectiveness Analyses: Second Panel on Cost-Effectiveness in Health and Medicine. JAMA.

[25] Cohen D.J., Reynolds M.R. (2008). Interpreting the results of cost-effectiveness studies. J. Am. Coll. Cardiol..

[26] Jia H., Lubetkin E.I. (2008). Estimating EuroQol EQ-5D scores from Population Healthy Days data. Med. Decis. Making.

[27] Yabroff K.R., Lund J., Kepka D., Mariotto A. (2011). Economic burden of cancer in the United States: Estimates, projections, and future research. Cancer Epidemiol. Biomarkers Prev..

[28] Pisu M., Henrikson N.B., Banegas M.P., Yabroff K.R. (2018). Costs of cancer along the care continuum: What we can expect based on recent literature. Cancer.

[29] Ellison A. 10 Largest Retail Pharmacies in America. https://www.beckershospitalreview.com/lists/10-largest-retail-pharmacies-in-america.html.

[30] Ranard K.M., Jeon S., Mohn E.S., Griffiths J.C., Johnson E.J., Erdman J.W. (2017). Dietary guidance for lutein: Consideration for intake recommendations is scientifically supported. Eur. J. Nutr..

[31] drugs.com Lycopene. https://www.drugs.com/npp/lycopene.html.

[32] NIH (2019). Omega-3 Fatty Acids–Fact Sheet for Health Professionals.

[33] Patterson R.E., Neuhouser M.L., Hedderson M.M., Schwartz S.M., Standish L.J., Bowen D.J. (2003). Changes in diet, physical activity, and supplement use among adults diagnosed with cancer. J. Am. Diet. Assoc..

[34] Greenlee H., Kwan M.L., Ergas I.J., Strizich G., Roh J.M., Wilson A.T., Lee M., Sherman K.J., Ambrosone C.B., Hershman D.L. (2014). Changes in vitamin and mineral supplement use after breast cancer diagnosis in the Pathways Study: A prospective cohort study. BMC Cancer.

[35] Bours M.J., Beijer S., Winkels R.M., van Duijnhoven F.J., Mols F., Breedveld-Peters J.J., Kampman E., Weijenberg M.P., van de Poll-Franse L.V. (2015). Dietary changes and dietary supplement use, and underlying motives for these habits reported by colorectal cancer survivors of the Patient Reported Outcomes Following Initial Treatment and Long-Term Evaluation of Survivorship (PROFILES) registry. Br. J. Nutr..

[36] Ravasco P. (2019). Nutrition in Cancer Patients. J. Clin. Med..

[37] Daly J.M., Redmond H.P., Gallagher H. (1992). Perioperative nutrition in cancer patients. JPEN J. Parenter. Enter. Nutr..

[38] McQuade R.M., Stojanovska V., Abalo R., Bornstein J.C., Nurgali K. (2016). Chemotherapy-Induced Constipation and Diarrhea: Pathophysiology, Current and Emerging Treatments. Front. Pharmacol..

[39] Bouabdallah I., D’Journo X.B. (2019). Risk factors of post-esophagectomy-induced malnutrition. J. Thorac. Dis..

[40] Ambrosone C.B., Zirpoli G.R., Hutson A.D., McCann W.E., McCann S.E., Barlow W.E., Kelly K.M., Cannioto R., Sucheston-Campbell L.E., Hershman D.L. (2020). Dietary Supplement Use During Chemotherapy and Survival Outcomes of Patients With Breast Cancer Enrolled in a Cooperative Group Clinical Trial (SWOG S0221). J. Clin. Oncol..

[41] Inoue-Choi M., Greenlee H., Oppeneer S.J., Robien K. (2014). The association between postdiagnosis dietary supplement use and total mortality differs by diet quality among older female cancer survivors. Cancer Epidemiol. Biomark. Prev..

[42] Costanzo S., De Curtis A., Di Castelnuovo A., Persichillo M., Bonaccio M., Pounis G., Cerletti C., Donati M.B., de Gaetano G., Iacoviello L. (2018). Serum vitamin D deficiency and risk of hospitalization for heart failure: Prospective results from the Moli-sani study. Nutr. Metab. Cardiovasc. Dis..

[43] Neuhouser M.L., Wassertheil-Smoller S., Thomson C., Aragaki A., Anderson G.L., Manson J.E., Patterson R.E., Rohan T.E., van Horn L., Shikany J.M. (2009). Multivitamin Use and Risk of Cancer and Cardiovascular Disease in the Women’s Health Initiative Cohorts. Arch. Intern. Med..

[44] Marian M.J. (2017). Dietary Supplements Commonly Used by Cancer Survivors: Are There Any Benefits?. Nutr. Clin. Pract..

[45] Paur I., Lilleby W., Bøhn S.K., Hulander E., Klein W., Vlatkovic L., Axcrona K., Bolstad N., Bjøro T., Laake P. (2017). Tomato-based randomized controlled trial in prostate cancer patients: Effect on PSA. Clin. Nutr..

[46] Peters U., Foster C.B., Chatterjee N., Schatzkin A., Reding D., Andriole G.L., Crawford E.D., Sturup S., Chanock S.J., Hayes R.B. (2007). Serum selenium and risk of prostate cancer-a nested case-control study. Am. J. Clin. Nutr..

[47] Lubinski J., Marciniak W., Muszynska M., Huzarski T., Gronwald J., Cybulski C., Jakubowska A., Debniak T., Falco M., Kladny J. (2018). Serum selenium levels predict survival after breast cancer. Breast Cancer Res. Treat..

[48] Harris H.R., Orsini N., Wolk A. (2014). Vitamin C and survival among women with breast cancer: A meta-analysis. Eur. J. Cancer.

[49] American Cancer Society American Cancer Society Guideline for Diet and Physical Activity. https://www.cancer.org/healthy/eat-healthy-get-active/acs-guidelines-nutrition-physical-activity-cancer-prevention/guidelines.html.

[50] Arends J., Bachmann P., Baracos V., Barthelemy N., Bertz H., Bozzetti F., Fearon K., Hütterer E., Isenring E., Kaasa S. (2017). ESPEN guidelines on nutrition in cancer patients. Clin. Nutr..

[51] Schloss J. (2016). Cancer treatment and nutritional deficiencies. Nutritional Deficiency.

[52] Ströhle A., Zänker K., Hahn A. (2010). Nutrition in oncology: The case of micronutrients (review). Oncol. Rep..

[53] Dreizen S., McCredie K.B., Keating M.J., Andersson B.S. (1990). Nutritional deficiencies in patients receiving cancer chemotherapy. Postgrad. Med..

[54] DiMartino L.D., Birken S.A., Mayer D.K. (2017). The Relationship Between Cancer Survivors’ Socioeconomic Status and Reports of Follow-up Care Discussions with Providers. J. Cancer Educ..

[55] ACL Administration for Community Living Profile of Older Americans. https://acl.gov/aging-and-disability-in-america/data-and-research/profile-older-americans.

[56] CDC (2019). NHANES Survey Methods and Analytic Guidelines.

[57] CDC NHANES Dietary Data 2011–2012. https://wwwn.cdc.gov/nchs/nhanes/search/datapage.aspx?Component=Dietary&CycleBeginYear=2011.

[58] AHRQ (2012). Medical Expenditure Panel Survey. https://meps.ahrq.gov/mepsweb/data_stats/download_data_files_detail.jsp?cboPufNumber=HC-155.

[59] AHRQ (2011). Medical Expenditure Panel Survey. https://meps.ahrq.gov/mepsweb/data_stats/download_data_files_detail.jsp?cboPufNumber=HC-147.

