# Supplementary material for: Cost-Effectiveness of Nutrient Supplementation in Cancer Survivors
Source: Cancers (Basel). 2021 Dec 14;13(24):6276. doi: 10.3390/cancers13246276 (PMC8699187; doi:10.3390/cancers13246276)
Supplement: Supplementary file 1 [file cancers-13-06276-s001.zip › cancers-1469892-supplementary.pdf]

Supplemental Tables

Table S-1. Odds of mortality according to supplementation use and previous hospitalizations

	OR	95% CI	*p*
No supplement use with hospitalizations	6.47	0.82, 50.82	0.07
No supplement use and no hospitalizations	1.15	0.26, 5.14	0.85
Supplement use with hospitalizations	6.04	1.00, 36.38	0.05
Supplement use and no hospitalizations	Ref	--	--

Abbreviations: OR, odds ratio; CI, confidence interval

p represents results of t-test or chi-square test of difference where applicable

Table S-2. Estimated average (per person) ten-year costs, by type and supplement use

Type	Source	Unit cost	No supplement use		Supplement use	
			$	%	$	%
**Hospital admission**	MEPS	4,030	4,030	2.5	4,030	2.5
**Healthcare**	MEPS and SEER					
**Initial**		60,000	60,000	37.7	60,000	36.9
**Continuing**		15,000	15,000	9.4	15,000	9.2
**End of Life**		80,000	80,000	50.3	80,000	49.2
**Supplements**	Market review	3,650			3,650	2.2
**Total**			159,030	100	162,680	100

Table S-3. Nutrients amount per daily dose/serving size and cost range

			Nutrients (amount per recommended daily dose/serving size)															
			Calcium (mg)	Folate (mcg)**	Iodine (mcg)	Lycopene (mcg)	Magnesium (mg)	Potassium (mg)	Total PUFA (g)	Selenium (mcg)	Lutein + Zeaxanthine (mcg)	Vitamin B6 (mg)	Vitamin B12 (mcg)	Vitamin C (mg)	Vitamin D (mcg)	Vitamin K (mcg)	Zinc (mg)	Dietary Fiber (g)
		RDA Female (Age 19+)	1000-1200	400	150	10,000-20,000	310-320	2,600	1.1	55	10,000 +2,000	1.3-1.5	2.4	75	15-20	90	8	21-25
		RDA Male (Age 19+)					400-420	3,400	1.6			1.3-1.7		90		120	11	30-38
		**Product Cost/day**																
**Multivitamins - $/day***	Nature Made Multi Complete	$0.09	160	400	150	---	100	---	---	70	---	2	6	180	25	80	15	---
	Nature Made Multi For Her	$0.16	250	600	150	---	100	---	---	70	---	2	6	180	25	80	15	---
	Nature Made Multi For Her 50+	$0.17	200	400	150	---	100	---	---	70	---	6	25	180	25	80	15	---
	Nature Made Multi For Him	$0.16	160	400	150	---	100	---	---	70	---	4	18	180	25	80	15	---
	Nature Made Multi For Him 50+	$0.16	160	400	150	---	100	---	---	105	---	6	25	180	25	80	15	---
	Centrum Adult	$0.09	200	400	150	---	50	80	---	55	---	2	6	60	25	25	11	---
	Centrum Women	$0.12	200	400	150	---	100	80	---	18	---	2	6	75	25	50	8	---
	Centrum Men	$0.12	210	200	150	600	100	80	---	100	---	2	6	90	25	60	11	---
	Centrum Silver Adult 50+	$0.12	220	400	150	300	50	80	---	19	250 (only lutein)	3	25	60	25	30	11	---
	Centrum Silver Women 50+	$0.12	300	400	150	---	100	80	---	22	300 (only lutein)	5	50	100	25	50	11	---
	Centrum Silver Men 50+	$0.12	210	300	150	600	75	80	---	21	300 (only lutein)	6	100	120	25	60	15	---
	Ocuvite Eye+Multi	$0.56	200	400	150	10,000	100	---	---	70	10,000 + 2,000	2	6	150	10	30	23	---
	One A Day Women	$0.12	380	400	150	---	---	---	---	27.5	---	1.7	6	75	25	25	8	---
	One A Day Men	$0.12	210	400	150	300	140	---	---	110	---	3	18	60	17.5	20	15	---
	Alive! Women's Energy	$0.28	260	240	150	---	50	---	---	55	100 (only lutein)	4.25	12	90	50	120	11	---
	Alive! Men's Energy	$0.28	200	400	150	600	100	80	---	140	100 (only lutein)	6	18	90	20	60	30	---
**Single-Nutrient Products - $/day***	Puritan's Pride Lycopene	$0.13	---	---	---	20,000	---	---	---	---	---	---	---	---	---	---	---	---
	Nature Made K-Gluconate	$0.08	---	---	---	---	---	90	---	---	---	---	---	---	---	---	---	---
	Nature Made Fish Oil	$0.32	---	---	---	---	---	---	1	---	---	---	---	---	---	---	---	---
	Puritan's Pride Softgels Lut+Ze	$0.12	---	---	---	---	---	---	---	---	20,000 + 800	---	---	---	---	---	---	---
	Walgreens Fiber Supplement	$0.07	---	---	---	---	---	---	---	---	---	---	---	---	---	---	---	3
	CVS Health Easy Fiber	$0.18	---	---	---	---	---	---	---	---	---	---	---	---	---	---	---	3
	Equate Sugar Free Fiber	$0.07	---	---	---	---	---	---	---	---	---	---	---	---	---	---	---	3
	Nature Made Fiber Gummies	$0.68	---	---	---	---	---	---	---	---	---	---	---	---	---	---	---	6

Abbreviations: mg, milligram; g, gram; mcg, microgram; Lut+Ze, lutein+zeaxanthine; RDA, Recommended Dietary All

**Folate amounts are folic acid values indicated on product labels.

*See products used for costs in the tables below

Costs were estimated using Pharmavite Brand, Nature Made's website, and through cost comparisons on the websites of retail pharmacies, CVS, Walmart, and Walgreens. These are the top three largest pharmacies in the U.S. based on number of stores, per Becker's Hospital Review.

Table S-4-5. Multivitamin and Single nutrient Products amount per daily dose/serving size and cost range

Multivitamin Products	Cost Range
Nature Made Multi Complete 130 tablets (1 tab/day)	$11.99
Nature Made Multi For Her 90 tablets (1 tab/day)	$13.99
Nature Made Multi For Her 50+ 90 tablets (1 tab/day)	$14.49
Nature Made Multi For Him 90 tablets (1 tab/day)	13.99
Nature made Multi For Him 50+ 90 tablets (1 tab/day)	$14.49
Centrum Adult 130 tablets (1 tab/day)	$11.29
Centrum Women 120 tablets (1 tab/day)	$13.99
Centrum Men 120 tablets (1 tab/day)	$13.99
Centrum Silver Adults 50+ 80 tablets (1 tab/day)	$9.99
Centrum Silver Women 50+ 100 tablets (1 tab/day)	$11.99
Centrum Silver Men 50+ 100 tablets (1 tab/day)	$11.99
Ocuvite Eye + Multi 60 tablets (2 tabs/day)	$16.79
One A Day Women 100 tablets (1 tab/day)	$11.79
One A Day Men 100 tablets (1 tab/day)	$11.79
Alive! Women 's Energy 50 tablets (1 tab/day)	$13.79
Alive! Men's Energy 50 tablets (1 tab/day)	$13.79

Single-Nutrient Products	Cost Range
Puritan's Pride Lycopene 20mg 60 softgels (1 softgel/day)	$7.99
Nature Made Potassium Gluconate 550mg 100 tablets (1 tab/day)	$8.29
Nature Made Ultra Omega-3 Fish Oil 1400mg 90 softgels (1 softgel/day)	28.99
Puritan's Pride Lutein 20mg + Zeaxanthine 800mcg 120 softgels (1softgel/day)	$14.99
Walgreens Fiber Supplement Powder 90 servings (1 serving/day)	$5.99
CVS Health Easy Fiber 125 servings (1 serving/day)	$22.79
Equate Sugar Free Fiber Supplement 125 servings (1 serving/day)	$9.34
Nature Made Fiber Gummies 90 gummies (3 gummies/day)	$20.49
